# Utility of the Pediatric Sleep Questionnaire and Pulse Oximetry as Screening Tools in Pediatric Patients with Suspected Obstructive Sleep Apnea Syndrome

**DOI:** 10.1155/2012/819035

**Published:** 2012-12-17

**Authors:** Jose A. Peña-Zarza, Borja Osona-Rodriguez de Torres, Jose Antonio Gil-Sanchez, Joan Figuerola-Mulet

**Affiliations:** Respiratory and Allergy Unit, Paediatrics Department, Son Espases University Hospital, 07013 Palma Mallorca, Balearic Islands, Spain

## Abstract

*Objective*. To assess the screening tools in snoring patients. *Material and Methods*. A retrospective review of data was conducted from children between 2 and 15 years old who were referred on suspicion of obstructive sleep apnea-hypopnea (OSAH) between June 2008 and June 2011. We excluded patients with significant comorbidities. Pediatric Sleep Questionnaire (PSQ), physical exam (PE), and pulse-oximetry data were collected and correlated with the results of the nightly polygraph at home. *Results*. We selected 98 patients. The 22-item version of the PSQ had sensitivity of 96% and specificity of 36.8%. The overall value of the clinic predictor of OSAH (PSQ and PE together) exhibited an increased specificity 57.6% with 94.6% of sensitivity. The nocturnal home oximetry method used alone was very specific, 92.1%, but had a lower sensitivity, 77.1%. The set of clinical assessment tools used together with pulse-oximetry screening provided excellent specificity 98.1% and a positive predictive value 94.1% globally. The performance of this screening tool is related with the severity of OSAH and accuracy is better in moderate and severe cases. *Conclusion*. The combination of clinical assessment and pulse-oximetry screening can provide a sufficient diagnostic approach for pediatric patients with suspected OSAH at least in moderate and severe cases.

## 1. Introduction

Snoring is a common symptom in children. It is estimated that 5–17% of children between the ages of 2 and 14 years snore, and that 2-3% within this age group suffer from obstructive sleep apnea-hypopnea (OSAH) [1–5]. Given the high prevalence of this group of symptoms, a series of tools have been designed in recent years with the purpose of identifying patients with greater clinical suspicion of OSAH in order to provide specialized treatment. Some of these tools include pediatric sleep questionnaires, such as the Pediatric Sleep Questionnaire (PSQ) designed by Chervin et al. [6] that has been validated in several studies [7], and the nocturnal pulse oximetry McGill scale described by Nixon et al. [8].

The current gold standard test for diagnosing obstructive sleep apnea-hypopnea (OSAH) is polysomnography (PSG), which is performed in a sleep laboratory. However, PSG is an expensive test that requires substantial resources, and is not available in all hospitals. As a diagnostic alternative, some centers used home cardiorespiratory polygraphy, which has already demonstrated its usefulness in the diagnosis of OSAH in children and adults [9, 10] and offers distinct advantages, such as not requiring hospital admission and being performed in the patient's normal bed, which allows for a more physiological relevant study, especially in the pediatric setting. Therefore, these benefits have led to its inclusion in the diagnostic algorithms of OSAH [11].

These various screening tools have been studied and compared to PSG individually, and none of them has achieved a sensitivity and specificity profile sufficient as individual methods for the diagnosis of OSAH. However, few studies have explored the utility of the diagnostic algorithm with these tools used in combination in children. Therefore, the aim of this study was to assess the correlation between clinical assessment (using the PSQ and physical examination), nocturnal pulse oximetry, or the combination of both approaches with the results of home nocturnal polygraphy.

## 2. Material and Methods

We performed a retrospective observational study of patients between the ages of 2 and 15 years who were referred to the pediatric pulmonology unit of our hospital on suspicion of OSAH between June 2008 and June 2011. The objective of the study was to assess the screening tools in snoring patients who lacked significant comorbidities. Therefore, patients with a known history of chronic lung disease, severe neurologic disease, or congenital heart disease were excluded from the study.

### 2.1. Clinical Assessment

Each patient underwent a full clinical assessment with a detailed history and physical examination. Anthropometric data were collected. The parent or guardian of each patient completed an abbreviated version of the PSQ containing 22 questions, and if 33% of more of the questions were affirmed, then the questionnaire was considered positive. The physical examination collected the following data related to OSAH: adenoid facies, nasal obstruction, and moderate or severe adenotonsillar hypertrophy when considering less than 75% space between pillars (grade III-moderate) or those were in direct contact (grade IV-severe). The examination was considered to be suggestive of OSAH if the patient had any of the three signs described above.

### 2.2. Pulse Oximetry

Home nocturnal pulse oximetry data were collected from a 3DI Pulsox Minolta pulse oximeter between the hours of 21:00 and 8:00 and considered valid for the study if they contained more than four hours of recordings. For staging of OSAH, a risk score was generated based on the scale described by Nixon et al. [8] Pulse oximetry was considered to be indicative of OSAH if the McGill score was greater than or equal to two. The results of pulse oximetry monitoring were used as a tool for prioritizing cardiorespiratory polygraphy, which was preferentially performed in patients who had pulse oximetry levels that were highly suggestive of OSAH.

### 2.3. Home Nocturnal Polygraphy

The polygraph was performed using a nocturnal home cardiorespiratory polygraph (Sleepscreen, Viasys Healthcare). The record was analyzed with Matrix Sleep Analysis software version 1.70.0.3 (Aequitron Medical; Minneapolis, MN, USA). The following variables were recorded: oronasal flow by cannula, thermistor and thoracoabdominal movements by induction bands, O_2_ saturation by pulse oximetry, heart rate, body position, and occurrence of snoring. In addition, the family members recorded evening events, such as waking, crying, and other variables. Data were considered valid in patients who had completed a minimum of four hours of full data collection without any artifacts. According to the criteria of the National Institutes of Health Consensus and American Academy of Sleep Medicine [12], obstructive apnea was defined as a decrease in oronasal flow equal to or greater than 90% for at least two respiratory cycles in the presence of respiratory effort, and obstructive hypopnea was defined as a drop in oronasal flow greater than 50% for at least two respiratory cycles accompanied by a 3% or greater decrease in SatO_2_. OSAH was diagnosed according to severity based on the apnea-hypopnea index as follows: mild between 3–5, moderate between 6–9, and severe > 10.

### 2.4. Statistical Analysis

We calculate the sensitivity, specificity, negative predictive value (NPV), and positive predictive value (PPV) of the different screening tools (PSQ, physical examination, and pulse oximetry) to diagnose OSAH. Standard formulas were used with reference to the results of the nightly Polygraphy at home. The receiver operating characteristic (ROC) curve was calculated from the questionnaires to assess the optimal cutoff value compared to the reference. A calculation was then performed to determine the sensitivity and specificity of the sum of the different diagnostic tools (combination of predictors) following the usual clinical algorithm. For statistical analyses, SPSS version 12.0 was used (IBM, USA). This study was approved by the Institutional Review Board (Ethical Committee of Balearic Island, Spain, IB 1770/12 PI).

## 3. Results

The records of 206 patients were analyzed from the pediatric pulmonology unit of our hospital. Forty-seven cases were excluded due to respiratory disease, severe cardiac, or neurological disorders. In 61 patients, the data were incomplete and not included. The remaining 98 patients used for analysis had a mean age of 7.2 years with a range of 2–15 years. Among them, 69% were male and 31% female. The mean body mass index (BMI) was 17.2 (±0.6). The characteristics of the cohort are described in Table 1. The sensitivity and specificity of the 22-item PSQ were 96% and 36.8%, respectively. The value of the area under the ROC curve was 0.789 and the number of positive responses from the test, which offered a better sensitivity and specificity profile, was 8 (33%) similar to that proposed by Chervin et al. [6].

The use of a physical examination (PE) as a predictor of OSAH showed a high sensitivity of 100% for all patients with positive nocturnal polygraphy and identified some clinical signs that were suggestive of OSAH (adenoid facies, nasal obstruction, or tonsillar hypertrophy) but with little specificity (39.7%; IC95 27.6–51.76%).

The PSQ and physical examination were part of the overall clinical assessment performed in the office by the physician. For this reason, we thought it would be interesting to measure the overall value of these tools as predictors of OSAH.

The use of nocturnal home pulse oximetry for monitoring sleep patterns is a minimally invasive test that has been validated and shown to provide an easy analysis for screening of OSAH in children [11]. We found that this method had a high specificity of 92.1% (IC95 85.4−98.7%) but a lower sensitivity of 77.1% (IC95 63.2–91.1%).

We analyzed the sensitivity and specificity of the different screening tools depending on severity of OSAH. The results are exposed in Table 2. We can see that PSQ and pulse oximetry have a very good sensitivity in moderate and severe OSAH but not in mild disease. 

**Figure 1:**
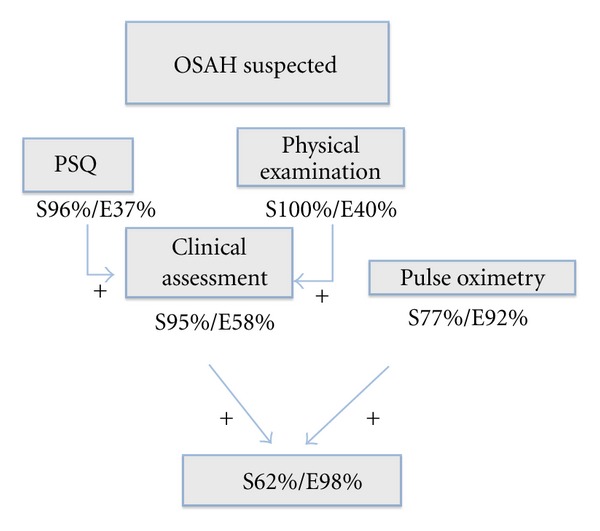
Assessment of screening tools related with home nocturnal polygraphy. S: sensitivity E: specificity PPV: positive predictive value NPV: negative predictive value.

**Figure 2:**
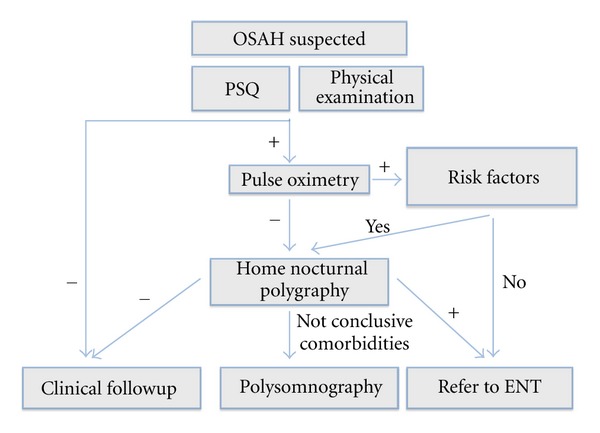
Proposed management algorithm in clinical practice. PSQ positive if 33% of affirmative answers. Physical examination: positive if we found adenoid facies, nasal obstruction, or tonsillar hypertrophy (III-IV). Pulse oximetry positive if Mc Gill index is II or greater. Risk factors: obesity, Down's syndrome, craniofacial abnormalities, neuromuscular disorders, sickle cell disease, or mucopolysaccharidosis.

Each of the screening tools described in this study demonstrated a different sensitivity and specificity profile for screening OSAH, and none of the methods alone was sufficient to reliably predict the outcome of the nightly polygraph. The global approach shows better results than each individual screening tool as we can see in Figure 1. Due to this results we propose a management algorithm described in Figure 2.

## 4. Discussion

The high prevalence of snoring as a clinical sign of a disorder in children and increased awareness among primary care pediatricians of the importance of early diagnosis of OSAH have caused an increase in examinations in the sleep unit of our hospital in recent years. As early as 1995, Carroll et al. [13] cautioned that clinical assessment alone was insufficient for the diagnosis of OSAH, and a meta-analysis published in 2004 by Brietzke et al. [14] reached the same conclusion. Since these findings, various tools have been designed to optimize the screening of these patients, including questionnaires and clinical scoring scales, which do not always have appropriate criteria for evaluation and standardization, as recently postulated by Spruyt and Gozal [15].

The 22-item PSQ is the most widely used method for assessing OSAH due to its psychometric properties and is specifically chosen because it has been effective in several languages [16]. The administration of this questionnaire to parents or guardians of pediatric patients with suspected OSAH in this study showed a high sensitivity (96%) but low specificity (37%), with a PPV of 40%. In the cases with negative results, we could rule out OSAH with almost certainty, but this required additional tools to confirm the diagnosis. The test was designed for patients between the ages of 2 and 18 years, the questions related to ADHD, such as “your child has difficulty organizing activities” or “your child is distracted by extraneous stimuli,” are almost always positive in patients younger than 4 years, and therefore affirmative answers are not necessarily indicators of a pathological behavior in this age group. In our cohort about 15% of patients are less than 4 years old. A previous study found an association between ADHD and OSAH [17], and therefore patients diagnosed with this disorder should always provide an adequate sleep history. However, a large group of patients with ADHD who do not exhibit OSAH could had a positive test due to these block of questions, specially the younger patients (less than 4 years) who are the 15% of our cohort.

Interestingly, our analysis of all of the tests showed high sensitivity (94.6.%) with a global clinical assessment. We found that patients where neither the history (supported by the PSQ) nor physical examination was suggestive of OSAH could be practically ruled out for a diagnosis of the disorder at least in moderate and severe cases. In addition, these patients could avoid other complementary examinations and would only receive a clinical followup, which would allow for monitoring of symptoms over time and evaluation of other possible causes, such as seasonal effects. In agreement with this idea, Gozal et al. [18] showed that variability in snoring and the severity of sleep-disordered breathing could be related to viral epidemics and the seasonality of different allergens.

Nocturnal pulse oximetry was also used as a screening tool in this patient population in a previous study by Broullitte that used the McGill score [11]. This method offers several advantages, such as easy performance, lower cost than PSG, and the easy identification of cluster patterns of desaturation associated with apnea and respiratory events. We observed a very good correlation between positive nocturnal pulse oximetry data and the polygraph results, with a specificity of 92%; however, we could not exclude an OSAH diagnosis based on a negative pulse oximetry result alone, and therefore further assessment was required. Based on these results and the proposed diagnostic scheme, we found that nearly 97% of patients who had a positive PSQ and pulse oximetry finding were subsequently diagnosed with OSAH, suggesting that this group of patients may not require an overnight polysomnography for diagnosis. From a practical standpoint, it seems reasonable to conclude that, although some authors stress the importance of a polysomnography before surgery or treatment of OSA in order to reduce surgical risk and facilitate the further treatment of the patient, approximately 20% of these patients have residual OSAH [19, 20]. This is an especially important preoperative assessment in patients of high-risk groups, such as those afflicted with obesity, Down's syndrome, craniofacial abnormalities, neuromuscular disorders, sickle cell disease, or mucopolysaccharidosis [21].

Few studies exist in the literature to date that have analyzed this set of screening methods for the diagnosis of OSAH in the pediatric setting. In 2006, Xu et al. [22] showed the utility of combining several predictive factors for diagnosing OSAH, including clinical history data (sleep apnea, mouth breathing, nocturnal enuresis, and occurrence of daytime naps), physical examination (tonsillar hypertrophy), and lateral neck radiography. The sensitivity of six selected predictors was shown to be 93.5% with a negative predictive value of 80%. These data suggest the use of this combination of predictors to properly screen and prioritize patients for complementary examinations, such as those with PSG findings that are suggestive of OSAH.

This study has several limitations. First, nightly cardiorespiratory polygraphy has been used for OSAH diagnosis, but the gold standard method for diagnosis of OSA is polysomnography [23, 24]. However, technical difficulties and the cost of PSG limit its use and make it impossible to be used in all patients with suspected sleep disorders. The sleep respiratory polygraphy technique is simpler and can be performed at the patient's home. This offers several advantages, such as the low cost and the convenience for the patient to sleep in their own bed in an accustomed environment, which is especially important in children [25]. It is known that desaturations are not frequent in children even after an obstructive apnea and that the common respiratory pattern of children with OSAH is represented by obstructive hypopnea. Although this technique may under diagnose hypopnea, it has been successfully evaluated for the diagnosis and screening of OSAH in both adults and children [26–29]. Given these limitations, cardiorespiratory polygraphy can be used for the diagnosis of OSAH in patients without other comorbidities, such as those included in this study. PSG could then be reserved for selected cases, such as those with suggestive symptoms but negative polygraph as well as those with other chronic diseases. Another limitation of our study was that all of the patients had been referred to the sleep unit of our hospital, which produces a sample bias, because patients are initially evaluated by their pediatrician and referred to the unit on suspicion of OSAH. This limits its applicability for the general population. 

Recently Kaditis et al. published a proposal algorithm for the diagnoses of OSAH in children [30], in the step one they recommended a “structured questionnaire” and they propose the pulsioximetry as an alternative tool in cases that overnight polysomnography is not available. However this group only consider another possibilities if PSG is not available but it is really difficult to make an overnight PSG in all the patients who have a clinical suggested of OSAH. An interview to ENT doctors shows that only 10% of patients who went under adenotonsillectomy had PSG prior to surgery and 65% of ENT make surgery depending on clinical findings even if PSG is normal [31].

In another publication Alonso Álvarez et al. propose [32] nocturnal cardiorespiratory polygraphy as an alternative even if PSG is available; if IAH measured by home polygraphy is superior of 5 they recommend to make adenotonsillectomy but if IAH is less than 5 they propose to make an overnight PSG to reject OSAH. 

Nowadays, the large number of patients with simple snoring and sleep respiratory disease and poor accessibility to PSG for diagnosis of OSAH requires that clinicians use diverse screening tools in a reasonable manner in order to optimize resources. Despite the limitations, our work presents data on the utility of each of these tools and provides a diagnosis scheme that can be used near the clinic and is accessible to most professionals. Further studies are needed to compare the proposed diagnostic algorithm with PSG in order to corroborate the results observed here.

## Figures and Tables

**Table 1:** Descriptive characteristics of cohort studied.

Total *n* cohort 98 patients	Total sample	AIH < 3 (51)	AIH 3–5 (24)	AIH 5–10 (14)	AIH > 10 (9)
Sex					
Male	69%	75% (38)	47% (11)	55% (8)	71% (6)
Female	31%	25% (13)	53% (13)	45% (6)	29% (3)
Physical examination					
Adenoid fascies	55%	11% (6)	32% (8)	65% (9)	88% (8)
Adenotonsillar hypertrophy (III-IV)	68%	34% (17)	45% (11)	75% (11)	77% (7)
Nasal obstruction	61%	30% (15)	51% (12)	72% (10)	77% (7)
Age					
<3 years old	11%	8% (4)	7% (2)	7% (1)	45% (4)
3–6 years old	53%	31% (16)	53% (13)	43% (6)	36% (3)
>6 years old	36%	61% (31)	40% (9)	50% (7)	19% (2)
BMI*					
BMI *z* score > 2SD	13%	12% (6)	16% (4)	14% (2)	10% (1)
AIH*					
<3	52%				
3–5 mild OSAH	24%				
5–10 moderate OSAH	15%				
>10 severe OSAH	9%				

BMI: body mass index, AIH: apnea/hipopnea index.

**Table 2:** Assessment of different screening tools depending on OSAH severity.

AIH-OSAS severity	PSQ	PE	CE	Pulse oximetry	Global evaluation
>3 mild OSAH	S 96%	S 100%	S 95%	S 77%	S 62%
	E 36.8%	E 40%	E 58%	E 92%	E 98%
>5 moderate OSAH	S 91%	S 100%	S 91%	S 100%	S 92%
	E 30.2%	E 14.9%	E 63%	E 80.1%	E 89%
>10 severe OSAH	S 100%	S 100%	S 100%	S 100%	S 100%
	E 22.7%	E 13.7%	E 61%	E 74.5%	E 86%

PSQ: Pediatric Sleep Questionnaire, PE: physical exam, CE: clinical evaluation (PSQ and PE both positive) global evaluation: CE and pulse oximetry positive AIH: apnea-hypopnea index.
